# Polypharmacy and risk of mortality among patients with heart failure following hospitalization: a nested case–control study

**DOI:** 10.1038/s41598-022-24285-4

**Published:** 2022-11-19

**Authors:** Sylvie Perreault, Mireille E. Schnitzer, Eliane Disso, Jakub Qazi, Laurie-Anne Boivin-Proulx, Marc Dorais

**Affiliations:** 1 Faculty of Pharmacy, University of Montreal, Case Postale 6128, Succursale Centre-Ville, Montreal, QC Canada; 2 Chaire Sanofi sur l’Utilisation des Médicaments de l’Université de Montréal, Montreal, Canada; 3 School of Public Health, University of Montreal, Montreal, QC Canada; 4 Faculty of Medicine, University of Montreal, Montreal, QC Canada; 5 CHUM Research Center, University of Montreal, Montreal, QC Canada; 6 StatSciences Inc., Notre-Dame-de-l’Île-Perrot, QC Canada; 7 Faculté de Pharmacie, Université de Montréal, Case Postale 6128, Succursale Centre-Ville, Montreal, QC H3C 3J7 Canada

**Keywords:** Cardiology, Health care

## Abstract

Heart failure (HF) is associated with morbidity, rehospitalization and polypharmacy. The incidence rate of mortality in HF patients with polypharmacy is poorly studied. We examine the association of polypharmacy with mortality risk in incident hospitalized HF patients with a primary diagnosis after discharge from the hospital using Quebec administrative databases, Canada from 1999 to 2015. Polypharmacy, cardiovascular (CV) polypharmacy and non-CV polypharmacy were respectively defined as exposure to ≥ 10 drugs, ≥ 5 CV drugs and ≥ 5 non-CV drugs within three months prior to the case or the control selection date. We conducted a nested case–control study to estimate rate ratios (RR) of all-cause mortality using a multivariate conditional logistic regression during one-year of follow-up. We identified 12,242 HF patients with a mean age of 81.6 years. Neither CV polypharmacy (RR  0.97, 95%CI 0.82–1.15) nor non-CV polypharmacy (RR  0.93, 95%CI 0.77–1.12) were associated with lower mortality risk. However, all polypharmacy (RR 1.31, 95%CI 1.07–1.61) showed an association with mortality risk. Myocardial infarction, valvular disease, peripheral artery disease, diabetes, major bleeding, chronic kidney disease, high comorbidity score, high Frailty score, hydralazine and spironolactone users were associated with increasing mortality risk, ranging from 15 to 61%, while use of angiotensin II inhibitors, beta-blockers, statins, anticoagulant, and antiplatelets were associated with lower risk, ranging from 23 to 32%.

## Introduction

Heart failure (HF) is associated with substantial morbidity and high rates of hospitalization in patients over the age of 65^1,2^. In outpatients with chronic HF, hospitalization is one of the most important predictors for increased mortality^1^. Unplanned admission in hospitals and rehospitalization also have an important impact on healthcare costs^3^.

A distinction should be made between outcomes in outpatients with chronic HF and patients with hospitalization for HF^1^. The prognosis of outpatients with HF has significantly improved in the last 20 years, given the use of angiotensin-converting enzyme inhibitors (ACEIs)/angiotensin II receptor blockers (ARBs), beta-blockers, mineralocorticoid receptor antagonists (MRAs) as a standard of care for patients with HF with reduced ejection fraction (HFrEF)^4,5^. In 2021 guideline, modern treatment of HFrEF with angiotensin receptor-neprilysin inhibitor (ARNI)/beta-blocker/MRA/sodium glucose transport 2 (SGLT2) inhibitor and diuretics are recommended^6^. In contrast, patients with hospitalization for HF continue to have a mortality rate approaching 8.8% and 36.3% at 30 days and one year, respectively^7^.

Published epidemiological data indicate that polypharmacy, defined as taking ≥ 10 drugs, is highly prevalent in older adults hospitalized for HF (between 84 and 95% from 2003 to 2014)^8^. The link between polypharmacy, which was mostly defined as taking ≥ 5 or ≥ 6 drugs, and the risk of all-cause mortality has been studied more broadly in elderly patients^9–11^. Most of these studies have concluded a positive association between polypharmacy and mortality. But to our knowledge, no observational studies have evaluated the association between polypharmacy and mortality in older adults with HF. Moreover, stratifying the polypharmacy by type of drugs may be helpful for the internal validity and to propose some target to be improved in HF management.

Thus, we examined the association between overall polypharmacy, cardiovascular (CV) polypharmacy and non-CV polypharmacy and a severe deleterious health outcome, such as mortality rate in the year following the first hospitalization for HF as a primary diagnosis from 1998 to 2015, using a nested case–control study.

## Methods

### Data source

We assembled a cohort from Med-Echo administrative databases (hospital discharges, medical services, and public drug insurance plans), administered by the *Régie de l’Assurance Maladie du Québec* (RAMQ)^12–14^. The databases were linked through encrypted personal health numbers. We have access to RAMQ administrative database, where no patient or physician identifiers were provided to the researchers; only scrambled identifiers were used throughout the study. Data access requests for the RAMQ databases are approved by the *Commission d’Accès à l’Information du Québec*. All efforts will be made to maintain the data confidentiality. Information from these databases provided a comprehensive picture of hospitalizations. The protocol received the approval of the University of Montreal Ethics Committee (*Comité d’éthique de la recherche en sciences et en santé* (CERSES) and all methods were performed in accordance with the relevant guidelines and regulations. The RAMQ covers all Quebec residents for the cost of physician visits, hospitalizations and procedures, and 94% of Quebec citizens aged 65 and older for the drug insurance plan^15^.

### Population definition

The initial cohort study was based on a random sample of 40% of the total cohort of patients aged 66 years and above from the RAMQ administrative databases from January 1999 and December 2015 in Quebec (The *Commission d’accès à l’information* does not authorize a sample over 40%). Using Med-Echo administrative databases, we identified patients admitted to the hospital with a primary diagnosis of HF (ICD-9 code “428.0, 428.1, 428.9” or ICD-10 code “I50.1, I50.9”) between January 1998 and December 2015 that were subsequently discharged from the hospital^16,17^. The patients also needed to have had no history of hospitalization for HF as primary diagnosis in the last 5-year period prior the hospitalization. To be eligible, the patients needed to have at least a 1-year history (prior to the cohort entry e.g., date of hospital discharge) of pharmaceutical services. The follow-up period ended in December 2016.

### Nested case–control study

We conducted a nested case–control study to estimate the rate ratio (RR) of all-cause mortality during one-year follow-up. All cases of all-cause mortality were identified by the date of death from RAMQ database and up to ten controls were randomly selected from the cohort based on the risk set for each case using density sampling^18^. Sampling for each control was selected in proportion to time contribution to the person-time at risk in the source population, which gives an unbiased estimate of the RR^18,19^. A hallmark of the risk set is that a cohort member who serves as control at one point time may later become a case, and a same cohort member may be selected as a control for more than one case^18,19^. Cases and controls were matched for age at the entry into the cohort, sex and for the date of the cohort entry.

### Exposure assessment

Total number of prescribed drugs was calculated from filled drug claims from the RAMQ drug insurance plan database. We defined three types of binary exposures: global polypharmacy (≥ 10 drugs vs < 10), CV polypharmacy (≥ 5 CV drugs vs < 5) and non-CV polypharmacy (≥ 5 non-CV drugs vs < 5). In previous studies, polypharmacy was typically defined as taking more than anywhere between five and nine drugs, while overall polypharmacy was defined as taking more than 10^20,21^. The drug exposure for each patient was assessed within the last three months prior to the date of case selection and the date of control selection. Moreover, the drug exposure was also evaluated by stratifying the period of three months prior to date of case as follows: (1) within day 1 and day 30 prior to the case, (2) within day 31 and day 60 prior to the case, (3) within day 61 and day 90 prior to the case.

### Covariates

We documented demographic data at cohort entry. We determined the associated morbidities by using diagnoses (using the specific ICD-9 or ICD-10 codes) made during the hospitalization for HF, such as hypertension, coronary artery disease, prior myocardial infarction, valvular heart disease, history of stroke, peripheral artery disease, hyperlipidemia, diabetes, chronic obstructive pulmonary disease, acute and chronic kidney disease, anemia, history of bleeding, liver disease, and cardiovascular disease (CVD) medical procedures (Table S1). The Charlson comorbidity index was considered as a co-morbidity metric, while frailty was assessed using the Elders Risk Assessment index which is typically used to stratify the two-year risk of total number of emergency room visits and hospitalizations for elderly individuals in the community^22,23^.

Data on aspirin claims were recorded but this count may be limited by the over-the-counter drug. We also obtained data on prescriptions for CV and non-CV drug after hospital discharge in the last three months prior the date of case selection or control selection. Drugs that may cause or exacerbate HF were identified from a scientific statement from the American Heart Association^24^. Other drugs that are potentially inappropriate in older adults were based on the American Geriatrics Society 2015 Updated Beers Criteria^25^.

### Statistical analysis

Descriptive analyses of sociodemographic and clinical characteristics were conducted for the entire cohort and according to the type of HF (left ventricular HF, and non-specific HF). Cumulative mortality incidence in 1-year of follow-up was done using Kaplan–Meier and log-rank test.

Conditional logistic regression models were developed to evaluate crude and adjusted rate ratios (RR) and 95% confidence intervals (CI) for all-cause mortality, as a primary outcome. Nested case–control analyses have been found to yield results that were similar to the Cox regression on the full cohort when studying time-dependent exposures, with the advantage of superior computational efficiency with the conditional logistic regression^26^. We provided a sensitivity analyses by reporting the demographic and clinical characteristics of the patients in the year prior to the cohort entry, including the hospitalization index. We also provided the assessment of covariates linked with mortality in the year prior to the cohort entry, including the hospitalization index.

Based on an increasing risk of death of 25% with a prevalence of polypharmacy (≥ 10 drugs) of 50%, we achieved a statistical power of 91.9% with a number of cases of 1000. Residuals from regression models were assessed for violations of the assumptions of multicollinearity or deviance. All the analyses were done using SAS version 9.4 (SAS Institute, Cary, North Carolina, USA).

### Consent to participate

No informed consent was needed for this study. This research was based on information extracted from user files without interactions with the research participants in accordance with the provisions of Section  125 of the Act respecting access to documents held by public bodies and the protection of personal information (L.R.Q c. A-2.1; public sector law) and Section  21 of the Act respecting the protection of personal information in the private sector (L.R.Q c. P-39.1; private sector law). Permission to receive the data used for this research was obtained by the *Commission de l’accès à l’information du Québec* and the research protocol received the approval of the University of Montreal Ethics Committee.

## Results

### Demographic and clinical characteristics

**Figure 1:**
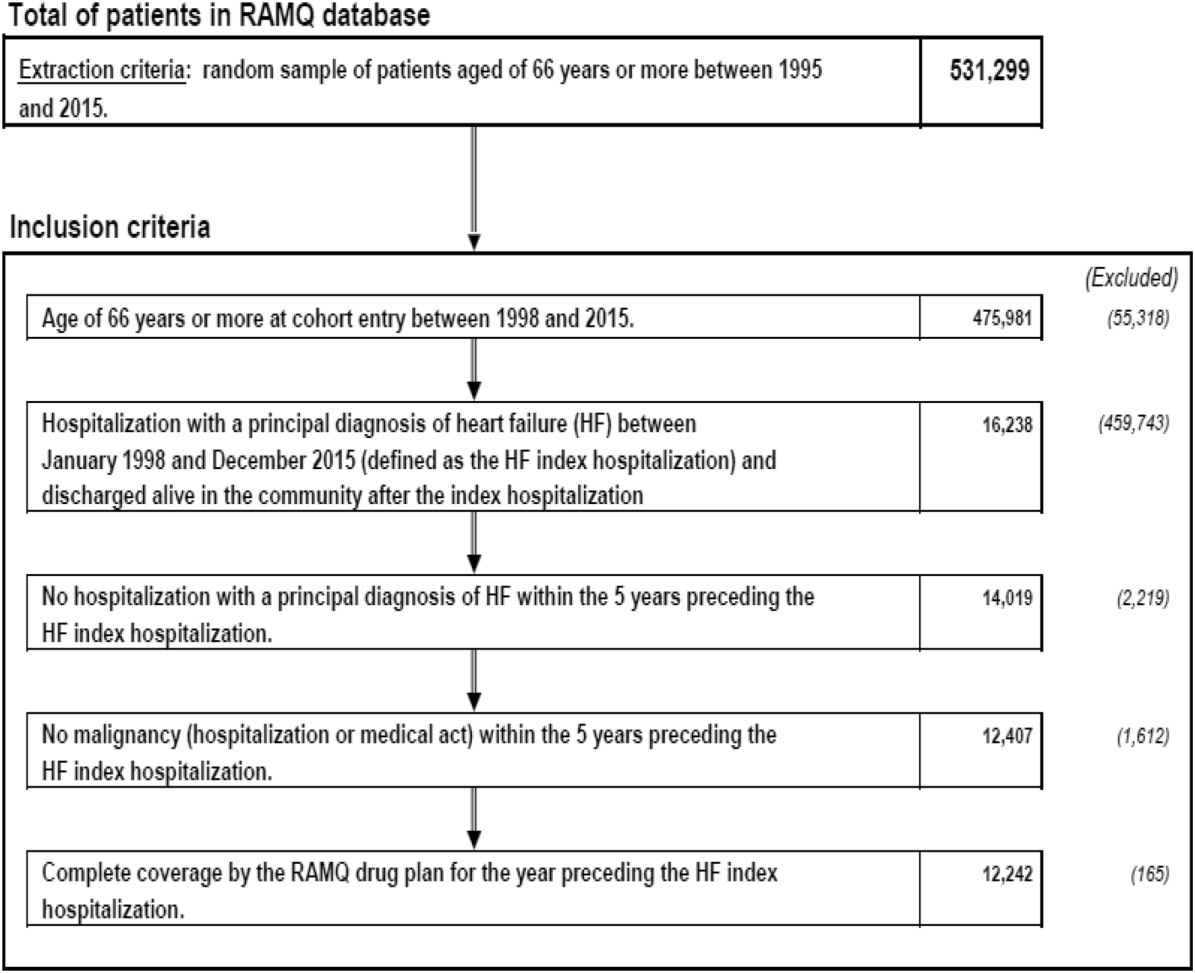
Study flow chart of a cohort of heart failure incident cases hospitalized between 1998 and 2015.

Among 16,238 patients who were hospitalized and had a diagnosis of HF as primary diagnosis of admission between January 1998 and December 2015, 12,242 patients met all selection criteria as shown in Fig. 1 and Table S2. The baseline of demographic and clinical characteristics of the cohort are shown in Table S3.

The full cohort had a mean age of 81.6 years and 45.3% were male (Table S3). As shown in Table S3, the most prevalent comorbidities presented by the patients in the cohort at baseline were coronary artery disease (38.7%) including myocardial infarction (18.4%) (total percentage of 57.1% for both together), hypertension (55.0%), cardiac arrhythmia (44.5%), chronic kidney disease (41.7%), diabetes (35.5%) and dyslipidemia (36.4%). Approximately half of the full cohort had left ventricular HF (50.1%), while non-specific HF accounted for 49.9%.

### Unadjusted cumulative incidence of mortality

The unadjusted cumulative incidence of mortality in 1-year of follow-up was 21.6%, which amounted to 2646 deaths (Table S2). As shown in Fig. S1, patients with CV polypharmacy presented no difference in mortality incidence rate relative to patients without CV polypharmacy. Conversely, the incidence rate of mortality in patients with non-CV polypharmacy was significantly greater than that in patients without non-CV polypharmacy (p < 0.0001). The incidence rate of mortality in patients with polypharmacy (≥ 10) was significantly greater than that in patients without polypharmacy (p < 0.0001). The incidence rate of mortality was significantly greater in patients with non-specific HF than those with left ventricular HF (log rank p < 0.0045).

### Polypharmacy exposure

In the nested case–control, 1530 cases were retained and were matched with 14,804 controls. As shown in Table 1, within three months preceding death, 73.7% of cases and 68.9% of controls had an overall polypharmacy. While CV polypharmacy was less prevalent in cases than controls (65.0% vs 70.3%, respectively), the opposite was true for non-CV polypharmacy (72.3% vs 66.7%) within the three-month period prior to selection. Meanwhile, as shown in Table 1, while CV polypharmacy decreased from 56.1% three months prior to selection to 23.3% one month prior to selection for cases, and from 60.9 to 60.4% for controls in the corresponding time periods. Similar reductions of non-CV polypharmacy were also observed e.g., non-CV polypharmacy from 59.4% 3 months prior to 28.4% 1 month prior to selection for cases and from 50.8 to 51.6% for controls.

**Table 1:** Drug use profile in the months preceding case occurrence in the nested case–control.

Cases (deaths): n = 1530	Within the 90 days prior to the case		Between day one and day 30 prior to the case		Between day 31 and day 60 prior to the case		Between day 61 and day 90 prior to the case	
Controls: n = 14,804								
	Cases	Controls	Cases	Controls	Cases	Controls	Cases	Controls
**Number of drugs used**								
Mean ± SD	12.9 ± 5.7	12.0 ± 4.8	5.0 ± 5.9	9.9 ± 4.5	9.7 ± 5.6	9.8 ± 4.4	10.4 ± 5.0	9.9 ± 4.4
Median (IQR)	13 (8)	12 (6)	2 (10)	10 (6)	10 (8)	10 (6)	10 (7)	10 (6)
≥ 5 drugs, N (%)	1425 (93.1)	14,277 (96.4)	657 (42.9)	13,294 (89.8)	1239 (81.0)	13,329 (90.0)	1351 (88.3)	13,372 (90.3)
≥ 10 drugs, N (%)	1128 (73.7)	10,194 (68.9)	405 (26.5%)	7826 (52.9)	826 (54.0)	7726 (52.2)	908 (59.4)	7734 (52.2)
**Number of CV drugs used**								
Mean ± SD	5.1 ± 2.2	5.4 ± 1.9	2.1 ± 2.6	4.8 ± 2.0	4.2 ± 2.4	4.8 ± 2.0	4.6 ± 2.2	4.8 ± 2.0
Median (IQR)	5 (3)	5 (3)	0 (4)	5 (2)	5 (3)	5 (2)	5 (3)	5 (2)
≥ 5 drugs, N (%)	995 (65.0)	10,404 (70.3)	356 (23.3%)	8943 (60.4)	774 (50.6)	8914 (60.2)	859 (56.1)	9014 (60.9)
≥ 10 drugs, N (%)	25 (1.6)	199 (1.3)	5 (0.3%)	56 (0.4)	8 (0.5)	50 (0.3)	6 (0.4)	58 (0.4)
**Number of non-CV medications used**								
Mean ± SD	7.7 ± 4.8	6.7 ± 4.1	2.9 ± 3.8	5.1 ± 3.5	5.5 ± 4.1	5.0 ± 3.4	5.8 ± 3.9	5.0 ± 3.4
Median (IQR)	7 (7)	6 (5)	1 (5)	5 (5)	5 (6)	5 (5)	5 (5)	5 (5)
≥ 5 drugs, N (%)	1106 (72.3)	9868 (66.7)	434 (28.4)	7638 (51.6)	835 (54.6)	7526 (50.8)	908 (59.4)	7515 (50.8)
≥ 10 drugs, N (%)	500 (32.7)	3301 (22.3)	101 (6.6)	1602 (10.8)	250 (16.3)	1544 (10.4)	276 (18.0)	1576 (10.7)

*SD* standard deviation, *IQR* interquartile range, *CV* cardiovascular.

### Nested case–control

Both cases and controls had a mean age of approximately 83 years (Table 2). Among cases vs controls, we noted more patients with coronary artery disease including myocardial infarction, valvular heart disease, peripheral artery disease, major bleeding, chronic kidney disease, chronic obstructive pulmonary disease, higher Charlson comorbidity index score ≥ 4, and higher Frailty score ≥ 16. Conversely, among controls, we observed more patients with cardiac arrhythmia, hypertension and dyslipidemia.

**Table 2:** Demographics, clinical characteristics, and healthcare use of case and control patients in the nested case–control.

Variables	Case (n = 1530)	Controls (n = 14,804)
Male, n (%)	727 (47.5)	7020 (47.4)
**Age (in years)**		
Mean ± SD	83.4 ± 6.7	83.2 ± 6.5
Median (IQR)	83.6 (9.6)	83.4 (9.4)
**Group, n (%)**		
66–74	184 (12.0)	1759 (11.9)
75–84	698 (45.6)	6969 (47.1)
85+	648 (42.4)	6076 (41.0)
**Charlson score, n (%)**		
< 4	321 (21)	4545 (30.7)
≥ 4	1209 (79.0)	10,259 (69.3)
**Frailty score, n (%)**		
< 4	111 (7.3)	1633 (11.0)
≥ 4–8	287 (18.8)	3417 (23.1)
≥ 9–15	480 (31.4)	4888 (33.0)
≥ 16	652 (42.6)	4866 (32.9)
**Comorbidites at cohort entry, n (%)**		
Coronary artery disease (excluding myocardial infarction)	618 (40.4)	5696 (38.5)
Myocardial infarction	313 (20.5)	2756 (18.6)
Cardiomyopathy	53 (3.5)	725 (4.9)
Any cardiac arrhythmia	636 (41.6)	6675 (45.1)
Atrial fibrillation	523 (34.2)	5305 (35.8)
Valvular heart disease	442 (28.9)	3844 (26.0)
Cerebrovascular disease	71 (4.6)	627 (4.2)
Peripheral artery disease	292 (19.1)	2126 (14.4)
Hypertension	754 (49.3)	8384 (56.6)
Dyslipidemia	476 (31.1)	5475 (37.0)
Diabetes and associated complications	555 (36.3)	5093 (34.4)
Major bleeding	183 (12.0)	1248 (8.4)
Major intracranial hemorrhage	29 (1.9)	171 (1.2)
Major gastrointestinal bleeding	157 (10.3)	1102 (7.4)
Chronic kidney disease	788 (51.5)	6043 (40.8)
Chronic kidney disease (CrCl < 30 mL/min/m^2^)	207 (13.5)	1326 (9.0)
Acute kidney disease	287 (18.8)	2413 (16.3)
Anemia	81 (5.3)	615 (4.2)
Chronic obstructive pulmonary disorder	549 (35.9)	4319 (29.2)
Pulmonary edema	20 (1.3)	179 (1.2)
Pneumonia	92 (6.0)	760 (5.1)
Liver disease	23 (1.5)	127 (0.9)
Rheumatic disease	141 (9.2)	1165 (7.9)
Gastroduodenal ulcers	27 (1.8)	359 (2.4)
Depression	32 (2.1)	265 (1.8)
Malign cancer	286 (18.7)	2499 (16.9)
**Medical procedures at cohort entry, n (%)**		
Percutaneous coronary intervention—stent/CABG	144 (9.4)	1343 (9.1)
Medical procedures for a defibrillator	73 (4.8)	700 (4.7)
**CVD drug use (in 3-month prior the case or control selection), n (%)**		
Diuretics	1382 (90.3)	13,503 (91.2)
Loop diuretics	1371 (89.6)	13,292 (89.8)
Metolazone	77 (5.0)	255 (1.7)
ACEIs/ARBs	763 (49.9)	9485 (64.1)
ACEIs	561 (36.7)	6756 (45.6)
ARBs	220 (14.4)	3068 (20.7)
Beta-blockers	921 (60.2)	9959 (67.3)
Metoprolol	405 (26.5)	4289 (29.0)
Carvedilol	78 (5.1)	657 (4.4)
Bisoprolol	377 (24.6)	4219 (28.5)
Other beta-blockers	77 (5.0)	942 (6.4)
Spironolactone or eplerenone	324 (21.2)	2628 (17.8)
Digoxin	331 (21.6)	3152 (21.3)
Hydralazine	110 (7.2)	531 (3.6)
Nitrates	697 (45.6)	5885 (39.8)
Statins	723 (47.3)	8399 (56.7)
Antiarrhythmic (amiodarone or propafenone)	169 (11.1)	1405 (9.5)
Warfarin	567 (37.1)	6179 (41.7)
DOAC	66 (4.5)	847 (5.8)
Antiplatelets	217 (14.2)	1982 (13.4)
Low-dose ASA	175 (11.4)	1748 (11.8)
**Antidiabetic agents (in 3-month prior the case or control selection), n (%)**	459 (30.0)	4615 (31.2)
Metformin	209 (13.7)	2297 (15.5)
Sulfonylurea	184 (12.0)	2060 (13.9)
Thiazolidinediones	8 (0.5)	68 (0.5)
DPP-4 inhibititors	29 (1.9)	344 (2.3)
Insulins	146 (9.5)	1431 (9.7)
**Potentially inappropriate drug for HF exacerbation (in 3-month prior the case or control selection), n (%)**		
Metformin	209 (13.7)	2297 (13.5)
Sulfonylurea	184 (12.0)	2060 (13.9)
Thiazolidinediones	8 (0.5)	68 (0.5)
DPP-4 inhibitors (saxagliptin/sitagliptin)	22 (1.4)	303 (2.1)
Calcium channel blockers	511 (33.4)	5844 (39.5)
Diltiazem/verapamil	149 (9.7)	1512 (10.2)
Nifedipine	35 (2.3)	549 (3.7)
Others	342 (22.4)	3945 (26.7)
Carbamazepine	8 (0.5)	54 (0.4)
Citalopram or escitalopram	140 (9.2)	1081 (7.3)
NSAIDs	27 (1.8)	479 (3.2)
Salbutamol	366 (23.9)	2559 (17.3)
Hydroxychlroroquine	21 (1.4)	140 (1.0)
**Other drug use (in 3-month prior the case or control selection), n (%)**		
Proton pump inhibitors	839 (54.8)	7742 (52.3)
Antidepressants agents	373 (24.4)	2824 (19.1)
Anticholinergics agents	42 (2.8)	267 (1.8)
Benzodiazepine	666 (43.5)	5704 (38.5)
Hospitalization in the month prior death	1178 (77.0)	2106 (14.2)

*SD* standard deviation, *IQR* interquartile range, *HF* heart failure, *CVD* cardiovascular disease, *CABG* coronary artery bypass grafting, *CrCl* creatinine clearance, *ACEIs* angiotensin-converting enzyme inhibitors, *ARBs* angiotensin II receptor blockers, *ASA* acetylsalicylic acid, *DOAC* direct oral anticoagulant, *DPP-4* dipeptidyl peptidase 4, *NSAIDs* non-steroidal anti-inflammatory drugs, *antiplatelets* clopidogrel, ticlopidine, prasugrel, ticagrelor.

As shown in Table 3, after adjustment, neither CV polypharmacy (adjusted RR 0.97, 95% CI 0.82–1.15), nor non-CV polypharmacy (adjusted RR 0.93, 95% CI 0.77–1.12) was related to a mortality risk, while all polypharmacy (adjusted RR 1.31, 95% CI 1.07–1.61) showed a significant increase in mortality risk.

**Table 3:** Rate ratios (RR; 95% confidence interval (CI)) for the associations between polypharmacy in the last 3 months and all-cause mortality.

	Crude RR (95% CI)	Adjusted RR (95% CI)
**Cardiovascular polypharmacy**		
< 5	Reference	Reference
≥ 5	0.79 (0.70–0.88)	0.97 (0.82–1.15)
**Non-cardiovascular polypharmacy**		
< 5	Reference	Reference
≥ 5	1.31 (1.16–1.47)	0.93 (0.77–1.12)
**Hyperpolypharmacy**		
< 10	Reference	Reference
≥ 10	1.27 (1.13–1.43)	1.31 (1.07–1.61)
**Type of HF**		
Ventricular HF	Reference	Reference
Non-specific HF	1.14 (1.03–1.27)	1.15 (1.03–1.28)
**Charlson score**		
< 4	Reference	Reference
≥ 4	1.67 (1.47–1.90)	1.20 (1.02–1.41)
**Frailty score**		
< 4	Reference	Reference
≥ 4–8	1.24 (0.99–1.55)	1.13 (0.89–1.44)
≥ 9–15	1.45 (1.17–1.79)	1.18 (0.93–1.50)
≥ 16	1.97 (1.60–2.43)	1.32 (1.03–1.69)
**Comorbidites at cohort entry (yes vs no)**		
Myocardial infarction	1.12 (0.99–1.28)	1.19 (1.01–1.40)
Cardiomyopathy	0.70 (0.53–0.93)	0.71 (0.53–0.95)
Any cardiac arrhythmia	0.87 (0.78–0.96)	0.78 (0.63–0.96)
Valvular heart disease	1.16 (1.03–1.30)	1.15 (1.02–1.30)
Peripheral artery disease	1.41 (1.23–1.61)	1.30 (1.13–1.51)
Hypertension	0.74 (0.67–0.83)	0.86 (0.77–0.96)
Dyslipidemia	0.77 (0.69–0.86)	0.85 (0.74–0.97)
Diabetes and associated complications	1.09 (0.97–1.21)	1.20 (1.01–1.44)
Major bleeding	1.48 (1.25–1.74)	1.23 (1.04–1.47)
Chronic kidney disease	1.54 (1.39–1.71)	1.30 (1.13–1.49)
Chronic kidney disease (CrCl < 30 mL/min/m^2^)	1.59 (1.36–1.86)	1.38 (1.16–1.64)
**Drug use (in 3-month prior the case or control selection)**		
ACEIs/ARBs	0.64 (0.55–0.75)	0.76 (0.65–0.89)
Beta-blockers	0.74 (0.66–0.82)	0.78 (0.69–0.89)
Spironolactone or eplerenone	1.25 (1.09–1.42)	1.21 (1.05–1.40)
Hydralazine	2.08 (1.68–2.58)	1.63 (1.30–2.05)
Nitrates	1.27 (1.14–1.41)	1.14 (1.00–1.29)
Statins	0.68 (0.62–0.76)	0.68 (0.60–0.78)
Warfarin or DOAC	0.79 (0.71–0.88)	0.76 (0.66–0.88)
Antiplatelets (including ASA)	0.95 (0.86–1.06)	0.86 (0.74–0.98)
Antidepressants agents	1.37 (1.21–1.55)	1.25 (1.07–1.47)

*RR* rate ratio, *CI* confidence interval, *CVD* cardiovascular disease, *CrCl* creatinine clearance, *ACEIs* angiotensin-converting enzyme inhibitors, *ARBs* angiotensin II receptor blockers, *HF* heart failure, *DOAC* direct oral anticoagulant, *ASA* acetylsalicylic acid, *antiplatelets* clopidogrel, ticlopidine, prasugrel, ticagrelor.

### Factors linked with mortality

As shown in Table 3, myocardial infarction, valvular heart disease, peripheral artery disease, diabetes and associated complications, prior major bleeding, chronic kidney disease, higher Charlson comorbidity index score and Frailty score were associated with an adjusted higher mortality risk, ranging from 15 to 32%. Meanwhile, any cardiac arrhythmias, cardiomyopathy, hypertension, and dyslipidemia were associated with a lower mortality risk, ranging from 14 to 29%.

We also observed specific drugs that were associated with lower rate of mortality. The use of ACEIs/ARBs, beta-blockers, statins, warfarin or direct oral anticoagulants (DOACs) and antiplatelet agents were associated with a significant 24%, 22%, 32%, 24% and 14% risk reduction of mortality, respectively (Table 3). On the other hand, hydralazine, spironolactone or eplerenone and antidepressants users were related to 63%, 21%, 25% and 25% greater mortality risk, respectively. The results of all covariates assessed for the link with the mortality risk are presented at Table S4.

### Sensitivity analysis

The demographic and clinical characteristics of the patients in the year prior to the cohort entry, including the hospitalization index, are reported in Table S5. The results are aligned to those observed at the cohort entry. And, as shown in Table S6, the results of the sensitivity analysis by including the covariates linked with mortality risk, in the year prior to the cohort entry including the hospitalization index, are well aligned with the results observed using the covariates at the cohort entry.

## Discussion

To our knowledge, this is the first real-world study investigating the association between polypharmacy and all-cause mortality in patients with HF, while also stratifying exposure by two types of polypharmacy. Neither CV polypharmacy and nor non-CV polypharmacy were associated to a significant mortality risk. However, overall polypharmacy showed a 31% higher risk of mortality. Myocardial infarction, valvular heart disease, peripheral artery disease, diabetes, major bleeding, chronic kidney disease, higher Charlson comorbidity index score and Frailty score, hydralazine, spironolactone or eplerenone and antidepressants users were related to a greater risk of mortality, ranging from 15 to 63%. Meanwhile, we observed that CV drug such as, ACEIs/ARBs, beta-blockers, statins, warfarin or DOACs and antiplatelet agents, were related to lower risk of mortality, ranging from 14 to 32%.

These findings are corroborated by current HF management guidelines, which hint at the protective nature of CV polypharmacy by emphasizing the value of combination therapies. For instance, triple HF therapy with ACEIs/ARBs, beta-blockers and MRAs or ARNI is mainstay in the management of HFrEF and was shown to improve survival^5,6^. Likewise, the Euro Heart Survey found that individual CV drug, such as beta-blockers (odds ratio (OR)   0.70, 95% CI 0.49–1.00) and ACEIs or ARBs (OR  0.60, 95% CI 0.46–0.80) reduced mortality in overall patients with HF^27^. Moreover, a recent meta-analysis found that ACEIs reduced all-cause mortality at 6 months (RR  0.76, 95% CI 0.66–0.87) and 12 months (RR  0.91, 95% CI 0.84–0.98) of follow-up^28^. Although, the relationships of cardiac arrhythmias, hypertension and dyslipidemia being linked with a lower mortality risk may be explained by two potential factors. First, it can be a healthy bias which arises when users of preventive drugs are healthier due to factors other than drug effects. And second, those observations may be linked with a more optimal of disease control management by physicians or health care professionals compared to those without those risk factors.

In this study, both hydralazine and spironolactone are typically used in older higher risk patients, such as those with severe or chronic hypertension and hypokalemia, respectively^29,30^. Again, hydralazine is mainly used in combination with nitrates in frailer patients who cannot tolerate ACEIs/ARBs because of their kidney function in HFrEF patients. And, in HF with preserved ejection fraction (HFpEF), MRAs could be used for the control retention in patients with repeated hospitalization. Although, the positive associations between hydralazine and the risk of mortality in our study could be explained by the fact that those patients are older higher risk patients with multiple comorbidities^31^. In fact, among hydralazine users in our cohort, only 20% of them were using ACEIs or ARBs. Conversely, the positive association between spironolactone and the risk of mortality is less intuitive, given that since the mean and median (interquartile range) dose of spironolactone used was 25 mg^31,32^.

Published epidemiological data indicate that polypharmacy is highly prevalent in older adults hospitalized for HF (between 84 and 95% from 2003 to 2014), as was the case in our study (93.1% to 96.4% in the three months preceding the case)^8^. But to our knowledge, no observational studies have evaluated the association between polypharmacy and mortality in older adults with HF. That being said, the relationship between polypharmacy, which was mostly defined as ≥ 5 or ≥ 6 drugs, and the risk of all-cause mortality has been studied more broadly in elderly patients^9–11^. Most of these studies have concluded a positive association between polypharmacy and mortality with relative risk values between 1.19 (95% CI 1.12–1.23) and 6.81 (95% CI 6.50–7.13)^9,11,33^, but some methodologic flaws were present in those studies. In our study, we were able to explain the positive association with polypharmacy by adjusting for a wide array of comorbidities.

However, in a British cohort study of older adults, polypharmacy (≥ 5 drugs) increased the two-year risk of mortality in both sexes (men hazard ratio (HR) = 1.94, 95% CI 1.59–2.37; women HR = 1.88, 95% CI 1.56–2.26) after adjusting for age, smoking status, disability and health conditions reported at baseline^9^. However, unlike our study, they evaluated their mortality risk outcome over a period of two years, and, most significantly, omitted important confounders, such as chronic kidney disease, from their models.

Recently, the study of Chang et al., using a propensity-score matched cohort study in the elderly (≥ 65 years), reported an increased mortality risk with polypharmacy (≥ 5 prescription drugs per day) after adjustment for age, sex, residential area, and the Charlson comorbidity index score (HR  1.25, 95% CI 1.24–1.25)^34^. Although this study was very well designed, controlling for a comorbidity index rather than the comorbidities themselves could have led to significant residual confounding by masking the effect of specific diseases^34^. Furthermore, the study of Chen et al., a cohort study evaluating the combined effect of polypharmacy and frailty on different clinical outcomes in the elderly consistently identified an association between polypharmacy (5–9 drugs) and polypharmacy (≥ 10 drugs) on all-cause mortality^33^. In this study, they noted that increased non-cancer mortality was observed with polypharmacy (5–9 drugs) and polypharmacy (≥ 10 drugs) in the model adjusted for age, sex, education, behavioral lifestyle, and associated morbidities, but the dose–response impacts were reduced after adjustment for the level of frailty, and a significant interaction was observed between polypharmacy (≥ 10 drugs) and level of frailty. Conversely to previous studies, these results suggested that polypharmacy only worsens mortality risk in the patients with little or no co-morbidities and decreases mortality risk in those with multimorbidity relative to the absence of polypharmacy.

Thus, it is important for any model assessing the association of polypharmacy and the risk of death to consider multimorbidity and frailty scores in the adjustment. Moreover, the period of polypharmacy assessment is crucial, since there is potential bias related to the fact that major changes in drugs prior to death may be in the clinical pathway of death, or drug intolerance or contraindication. For example, a hospitalization in the month prior to death is in the clinical pathway of death and may result in drug changes. Bias may arise due to ‘the fact that drug exposure is often reduced for patients with reduced life expectancy or drug intolerance related to the blood pressure level or altered kidney function or related to a recent critical hospitalization’. Both situations may result in overadjustment bias due to controlling for an intermediate variable on a causal pathway between the exposure and outcome^35^.

The adequate selection of cases and controls, definition of drug exposure, and control of confounding, particularly confounding by indication, which is common to polypharmacy studies, underlie the strengths and weaknesses of our study^36^. One of our study’s primary advantages is the precision and completeness of our data source. In addition to our large sample size, our data source gave us access to a wide array of demographic and clinical variables. Furthermore, the study design, a nested case–control study, favored an unbiased selection of controls, while our indication-specific definition of polypharmacy made it easier for us to adequately describe its association with mortality. We controlled for confounding by matching cases and controls by age, sex, and time since hospitalization, and by adjusting for a long list of confounders in the regression models. Lastly, the adjustment for at least five comorbidities may allow for some control of indication bias as suggested by a systemic review of the influence of health outcomes in polypharmacy studies^36^.

Our study was also subject to limitations. First, our estimation of drug exposure is constrained by filled drug claims data. Second, our inability to measure severity of illness preceding death might have allowed for residual confounding. Third, unmeasured demographic and clinical variables such as socio-economic status, blood pressure, hemoglobin, glycated hemoglobin, exact estimated glomerular filtration rate, ventricular function and smoking status could be another source of residual confounding. Four, given modern treatment of HFrEF with ARNI/beta-blocker/MRA/SGLT2 and diuretic, recent guideline modification that take time to be implemented and it takes time to have a sufficient sample size following the implementation to include to category of treatment in the analyses. Five, the lack of information on the severity of HF and associated morbidity is as an important residual confounder. Lastly, our results may not be generalizable to other groups such as, non-hospitalized older adults with HF and other ethnic groups, as our population was mostly white.

The ageing of the population is linked with polypharmacy and frailty, both resulting in adverse health outcome and mortality rates, highlighting the need to decrease needless polypharmacy^37^. While deprescribing may reduce inappropriate drug use but its impact on clinical outcomes remains unclear^38^. Finally, the management of cardiovascular and non-cardiovascular comorbidities in HF should also be based on appropriate treatment selection and recent advances^39,40^.

## Conclusion

Our study was the first to characterize the relationship between two types of polypharmacy and mortality in patients with HF. Our findings suggest that polypharmacy was related to a greater susceptibility to all-cause mortality, but some CV drugs were associated with a lower mortality risk. Future work will involve investigating this association for patients with HFpEF and HFrEF.

## Supplementary Information

Supplementary Information.

## Data Availability

All data generated or analysed during this study are included in this published article and its supplementary information file.
