# Supplementary material for: Polypharmacy and risk of mortality among patients with heart failure following hospitalization: a nested case–control study
Source: Sci Rep. 2022 Nov 19;12:19963. doi: 10.1038/s41598-022-24285-4 (PMC9675839; doi:10.1038/s41598-022-24285-4)
Supplement: Supplementary file 1 — Supplementary Information. [file 41598_2022_24285_MOESM1_ESM.pdf]

Supplementary Information

Polypharmacy and Risk of Mortality Among Patients with Heart Failure Following Hospitalization: A Nested Case-control Study

Scientific Reports

Sylvie Perreault[1*], Mireille E Schnitzer[1,2], Eliane Disso[2], Jakub Qazi[1], Laurie-Anne Boivin-Proulx[3,4], Marc Dorais[5]

[1] Faculty of Pharmacy, University of Montreal, Montreal, Quebec, Canada

[2] School of Public Health, University of Montreal, Montreal, Quebec, Canada

[3] Faculty of Medicine, University of Montreal, Montreal, Quebec, Canada.

[4] CHUM Research Center, University of Montreal, Montreal, Quebec, Canada

[5] StatSciences Inc., Notre-Dame-de-l'Île-Perrot, Quebec, Canada

***Corresponding Author:** Sylvie Perreault

Chaire Sanofi sur l'utilisation des médicaments

Faculté de Pharmacie, Université de Montréal, Case Postale 6128, Succursale Centre-Ville, Montréal, Québec, Canada, H3C 3J7

Centre de recherche en santé publique (CReSP)**,** partenaire CIUSSS du Centre-Sud-de-l'Île-de-Montréal et l'Université de Montréal, Montreal, Quebec, Canada

Tel: +1 (514) 343-6111 ext. 3149

Fax: +1 (514) 343-6120

E-mail: sylvie.perreault@umontreal.ca

Description of data sources: **RAMQ database**

The cohort is coming from the Med-Echo administrative databases and the "*Régie de l'Assurance Maladie du Québec*" (RAMQ). The Med-Echo administrative databases, which store data on hospital discharges, and RAMQ databases, which store data on medical services, and public drug plans, and all those databases are managed by the RAMQ.[1-4] The databases were linked using encrypted health insurance numbers. The information from these databases provide a comprehensive picture of the status of hospital admissions, medical services and public drug plans.

Data were collected from the RAMQ and Med-Echo databases, which administer public healthcare insurance programs in the province of Quebec, Canada. The Med-Echo database gathers information on acute care hospitalizations, such as date of admission, length of stay, primary and up to 15 secondary diagnoses. The RAMQ data were extracted from three databases. The beneficiary database lists age, gender, socioeconomic status, and date of death. The medical services file contains claims for all inpatient and ambulatory services and diagnostic codes are classified according to the International Classification of Diseases, 9th Revision (ICD-9). All surgical procedures codes follow the Canadian classification of diagnostic, therapeutic and surgical procedures.[5] The pharmaceutical database provides data on delivered medication in community pharmacies such as the date of filling, name of the drug, dose, quantity, dosage form, and duration of therapy. The RAMQ covers all Quebec residents for the cost of physician visits, hospitalizations and procedures, and 94% of Quebec citizens aged 65 and older for the drug plan.[1,3]

1. Tamblyn, R., Lavoie, G., Petrella, L. & Monette, J. The use of prescription claims databases in pharmacoepidemiological research: the accuracy and comprehensiveness of the prescription claims database in Quebec. *J. Clin. Epidemiol*. **48**, 999-1009 (1995).
2. Eguale, T., Winslade, N., Hanley, J.A., Buckeridge, D.L. & Tamblyn, R. Enhancing pharmacosurveillance with systematic collection of treatment indication in electronic prescribing: a validation study in Canada. *Drug Saf*. **33**, 559-567 (2010).
3. Wilchesky, M., Tamblyn, R.M. & Huang, A. Validation of diagnostic codes within medical services claims. *J. Clin. Epidemiol*. **57**, 131-141 (2004).
4. Tamblyn, R., Reid, T., Mayo, N., McLeod, P. & Churchill-Smith, M. Using medical services claims to assess injuries in the elderly: sensitivity of diagnostic and procedure codes for injury ascertainment. *J. Clin. Epidemiol*. **53**, 183-194 (2000).
5. Régie de l'assurance maladie du Québec. Rapport annuel de gestion 2005-2006. Quebec: Régie de l'assurance maladie du Québec (2006).

ICD-9, International Classification of Diseases, 9th Revision; RAMQ, *Régie de l'Assurance Maladie du Québec*.

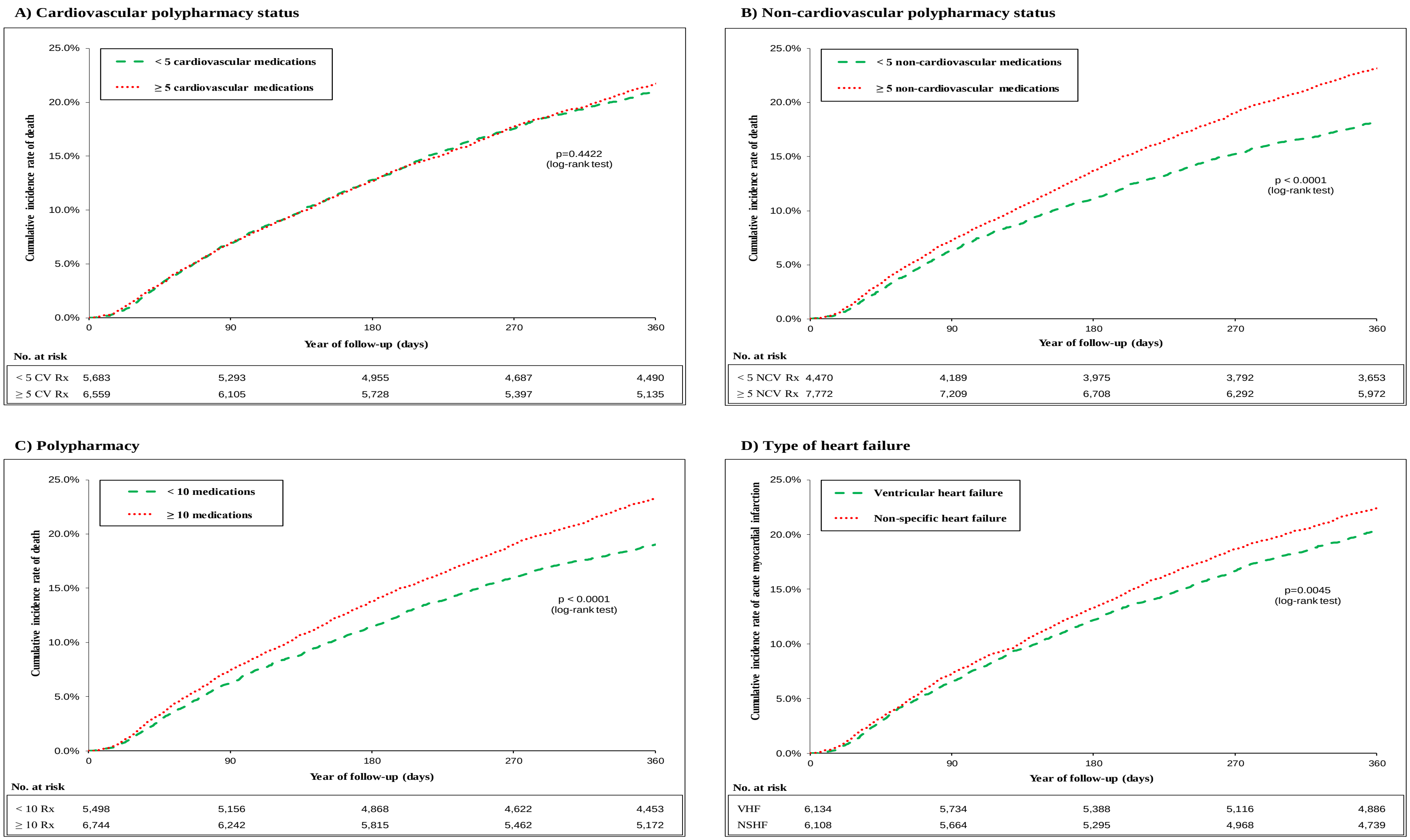

A) No. at risk

< 5 CV Rx	5,683	5,293	4,955	4,687	4,490
≥ 5 CV Rx	6,559	6,105	5,728	5,397	5,135

B) No. at risk

< 5 NCV Rx	4,470	4,189	3,975	3,792	3,653
≥ 5 NCV Rx	7,772	7,209	6,708	6,292	5,972

C) No. at risk

< 10 Rx	5,498	5,156	4,868	4,622	4,453
≥ 10 Rx	6,744	6,242	5,815	5,462	5,172

D) No. at risk

VHF	6,134	5,734	5,388	5,116	4,886
NSHF	6,108	5,664	5,295	4,968	4,739

Figure S1. Cumulative mortality incidence in one-year of follow-up by: A. cardiovascular polypharmacy status; B. non-cardiovascular polypharmacy status; C. Polypharmacy status; D. Subtype of heart failure.

CV, cardiovascular; NCV, non-cardiovascular; VHF, ventricular heart failure; NSHF, non-specific heart failure.

Comorbidities	ICD-9 codes	ICD-10 codes
Left ventricular heart failure	428.1	I50.1
Non specific heart failure		
Non-specific congestive heart failure	428.0	I50.0
Non-specific heart failure	428.9	I50.9
Ischemic cardiopathy	410, 411, 413, 414, 414.0, 414.1, 414.8, 414.9	I20, I20.0, I20.1, I20.8, I20.9 I23, I24, I25, I25.1, I25.3, I25.4, I25.5, I25.8, I25.9
Myocardial infarction	412	I22, I23, I25.2
Cardiomyopathy	425.4, 425.11, 425.18, 425.0, 425.3, 425.4, 425.5, 425.9, 425.2, 425.4, 425.4, 425.9, 425.8	I42.0, I42.1, I42.2, I42.3, I42.4, I42.5, I42.6, I42.7, I42.8, I42.9, I43, I43.0, I43.1, I43.2, I43.8
Cardiac arrhythmia	427, 427.0, 427.1, 427.2, 427.4, 427.5, 427.6, 427.8	I46, I46.0, I46.1, I46.9, I47.0, I47.1, I47.2, I47.9, I49.0, I49.1, I49.2, I49.3, I49.4, I49.5, I49.8, I49.9
Atrial fibrillation	427.3	I48
Valvular heart disease	394, 394.0, 394.1, 394.2, 394.9, 395.0, 395.1, 395.2, 395.9, 396.0, 396.1, 396.2, 396.3, 396.8, 396.9, 397, 397.0, 397.1, 397.9, 424.0, 424.1, 424.3, 424.90, 421.1	I05.0, I05.1, I05.2, I05.8, I06.0, I06.1, I06.2, I06.8, I06.9, I08.0, I08.0, I08.0, I08.0, I08.8, I08.9, I07.1, I07.2, I07.8, I09.89, I09.1, I34, I34.0, I34.1, I34.2, I34.8, I34.9, I35, I35.0, I35.1, I35.2, I35.8, I35.9, I37, I37.0, I37.1, I37.2, I35.8, I35.9, I38, I39, I39.0, I39.1, I39.2, I39.3, I39.4, I39.8
Cerebrovascular diseases	430, 431, 432, 432.0, 432.1, 433, 433.0, 433.1, 434, 434.00, 433.2, 434.01, 435, 434.01, 433.3, 436, 437, 437.0, 437.1, 438, 437.2, 437.3, 432.9, 433.8, 437.4, 433.9, 437.5, 434.9, 437.6, 437.8, 434.11, 437.9, 433.21, 433.21, 433.11, 433.91, 433.21, 434.91, 433.31, 433.81	I60, I61, I62, I62.1, I62.0, I62.9, I65, I65.1, I65.2, I65.0, I65.3, I65.8, I65.9, I66, I66.x, I63.30, G45.8, G45.9, I67.89, I67, I67.2, I67.3, I67.4, I67.1, I67.7, I67.5, I67.6, I67.89, I67.9, I67.0, I69, I63.0, I63.1, I63.2, I63.3, I63.4, I63.5, I63.8, I63.9, I67, I68
Peripheral artery disease	440, 440.0, 440.1, 440.2, 440.8, 440.9, 441, 441.0, 441.1, 441.2, 441.3, 441.4, 441.5, 441.6, 441.7, 441.9, 442, 442.0, 442.1, 442.2, 442.3, 442.8, 442.9, 443, 443.0, 443.1, 443.8, 443.9, 444, 444.0, 444.1, 444.2, 444.8, 9, 446, 446.5, 446.7	I70, I70.0, I70.1, I70.2, I70.8, I70.9, I71, I71.0, I71.1, I71.2, I71.3, I71.4, I71.5, I71.6, I71.8, I71.9, I72, I72.0, I72.1 I72.2, I72.3, I72.8, I72.9, I73, I73.0, I73.1, I73.8, I73.9, I74, I74.0, I74.1, I74.2, I74.3, I74.4, I74.5, I74.8, I74.9, M30, M31.4, M31.5, M31.6
Hypertension	401.0, 401.1, 401.9	I10.0
Cardiomyopathy et renal failure	404	I13
Hypertensive cardiopathy	402	I11.0
Dyslipidemia	272, 272.0, 272.1, 272.2, 272.3, 272.4, 272.5, 272.6, 272.7, 272.8, 272.9	E78, E78.0, E78.1, E78.2, E78.3, E78.4, E78.5, E78.6, E78.8, E78.9
Diabetes and associated complications	250, 357.2, 362.0, 366.41	E10, E12, E13, E14

Major bleeding (including intracranial hemorrhage, gastrointestinal bleeding and other major bleeding)	430, 431, 432.x, 852.x, 853.x, 456.1, 530.7, 531.0x, 531.2x, 531.4x, 531.6x, 532.0x, 532.2x, 532.4x. 532.6x, 533.0x, 533.2x, 533.4x, 533.6x, 534.0x, 534.2x, 534.4x, 534.6x, 535.1, 537.83, 578.0, 562.02, 562.03, 562.12, 562.13, 569.3x, 569.85, 578.1x, 578.9, 599.7, 786.3x, 379.23, 626.2x, 280.0, 285.1, 285.9, 719.1x, 423.0, 568.8, 459.0x, 285.1x, 996x, 997x, 998x, 999x, 8602, 8603, 8604, 8605, 851x, 920x, 921x, 922x, 923x, 924x.	I60, I61, I62, S06.3, S06.4, S06.5, S06.6, I85.0, K22.6, K25.0, K25.2, K25.4, K25.6, K26.0, K26.2, K26.4, K26.6, K27.0, K27.2, K27.4, K27.6, K28.0, K28.2, K28.4, K28.6, K29.0, K31.811, K92.0, K57.11, K57.13, K57.31, K57.33, K62.5, K55.21, K92.1, K92.2, R31, R04.2, R04.89, R04.9, H43.13, N92.0, D50.0, D62, D64.9, M25.0x, I31.2, K66.1, R58.0, D62, S271x, S272.x
Major intracranial hemorrhage	430, 431, 432.x, 852.x, 853.x	I60, I61, I62, S06.3, S06.4, S06.5, S06.6
Major gastrointestinal bleeding	456.1, 530.7, 531.0x, 531.2x, 531.4x, 531.6x, 532.0x, 532.2x, 532.4x. 532.6x, 533.0x, 533.2x, 533.4x, 533.6x, 534.0x, 534.2x, 534.4x, 534.6x, 535.1, 537.83, 578.0, 562.02, 562.03, 562.12, 562.13, 569.3x, 569.85, 578.1x, 578.9	I85.0, K22.6, K25.0, K25.2, K25.4, K25.6, K26.0, K26.2, K26.4, K26.6, K27.0, K27.2, K27.4, K27.6, K28.0, K28.2, K28.4, K28.6, K29.0, K31.811, K92.0, K57.11, K57.13, K57.31, K57.33, K62.5, K55.21, K92.1, K92.2
Other major bleeding	599.7, 786.3x, 379.23, 626.2x, 280.0, 285.1, 285.9, 719.1x, 423.0, 568.8, 459.0x, 285.1x, 996x, 997x, 998x, 999x, 8602, 8603, 8604, 8605, 851x, 920x, 921x, 922x, 923x, 924x.	R31, R04.2, R04.89, R04.9, H43.13, N92.0, D50.0, D62, D64.9, M25.0x, I31.2, K66.1, R58.0, D62, S271x, S272.x
Chronic kidney disease	249.4, 250.4, 403, 404, 581.81, 582, 583, 583.81, 583.9, 585, 586, 587, 588, 589, 590.0	E08.21, E11.2, N03, N05, N05.9, N08, N17.1, N17.2, N18, N19, N25, N26.9, N27, I12, I13
Chronic kidney disease (creatinine clearance ≤ 30 mL/min/m^2)	585 diagnostic code within 2 to 5 years and/or specific drug use within 2 years (antihypertensive agent, hyperlipidemic agent, oral antidiabetic, calcium carbonate ≥ 1500 mg/day, calcitriol ≥ 0.25µg 3/week or ≥ 0.75 µg/week or ≥ 3 µg/30 days, doxercalcifrrol ≥ 2.5µg/day or ≥ 17.5 µg/week or ≥ 75 µg/30 days, alfacalcidol ≥ 0.25µg 3/week or ≥ 0.75 µg/week or ≥ 3 µg/30 days, sevelamer, cinacalcet, lanthanum) and nephrologist visit ≥4 within 2 to 5 years	N18 diagnostic code within 2 to 5 years and/or specific drug use within 2 years (antihypertensive agent, hyperlipidemic agent, oral antidiabetic, calcium carbonate ≥ 1500 mg/day, calcitriol ≥ 0.25µg 3/week or ≥ 0.75 µg/week or ≥ 3 µg/30 days, doxercalcifrrol ≥ 2.5µg/day or ≥ 17.5 µg/week or ≥ 75 µg/30 days, alfacalcidol ≥ 0.25µg 3/week or ≥ 0.75 µg/week or ≥ 3 µg/30 days, sevelamer, cinacalcet, lanthanum) and nephrologist visit ≥4 within 2 to 5 years
Acute kidney disease	580, 581.xx, 584.xx	N00.x, N04.x, N08.x, N17.x
Chronic obstructive pulmonary disorder	490.xx, 491.0, 491.1, 491.2, 491.8, 491.9, 492.0, 492.8, 493.0, 493.1, 493.2, 493.9, 494.xx, 496.xx	J40, J41.x, J42, J43.x, J44.x, J45.x
Pulmonary edema	428.1, 518.4	I50.1, J81
Pneumonia	480-488	J09, J10, J11, J12, J13, J14, J15, J16, J17, J18

Liver disease	570.xx, 571.0, 571.1, 571.2, 571.3, 571.4, 571.5, 571.6, 571.8, 571.9	K70.xx, K71.xx, K72.xx, K73.xx, K74.xx
Rheumatic disease	274.xx, 710.0, 714.xx	M05.xx, M14.xx, M32.x
Gastroduodenal ulcers	530.x, 530.0, 530.3, 530.4, 530.5, 530.6, 530.7, 530.9, 578.0, 578.1, 578.9	K22.0, K22.2, K22.3, K22.4, K22.5, K22.6, K22.9, K920, K921, K922
Depression	296.2, 296.3, 296.6, 296.8, 296.8, 296.99, 298.x, 300.4, 301.10, 301.12, 301.13, 311.xx	F32.xx, F33.xx, F34.xx, F38.xx, F39.xx

Table S1. Diagnostic codes of comorbidities.
Roy, L. *et al.* Agreement between administrative database and medical chart review for the prediction of chronic kidney disease G category. *Can. J. Kidney Health Dis.* **7**, 2054358120959908 (2020)

**Variables**	**Case (n = 1,530)**	**Case excluded < three-month of drug exposure (n = 1,116)**
**Male**, n (%)	727 (47.5)	472 (42.3)
**Age** (in years)		
Mean ± SD	83.4 ± 6.7	85.3 ± 7.3
Median (IQR)	83.6 (9.6)	85.6 (10.3)
**Group**, n (%)		
66–74	184 (12.0)	100 (9.0)
75–84	698 (45.6)	424 (38.0)
85 +	648 (42.4)	592 (53.0)
**Charlson score**, n (%)		
< 4	321 (21)	219 (19.6)
≥ 4	1,209 (79.0)	897 (80.4)
**Frailty score**, n (%)		
< 4	111 (7.3)	60 (5.4)
≥ 4–8	287 (18.8)	204 (18.3)
≥ 9–15	480 (31.4)	328 (29.4)
≥ 16	652 (42.6)	524 (46.9)
**Comorbidites at cohort entry**, n (%)		
Coronary artery disease (excluding myocardial infarction)	618 (40.4)	429 (38.4)
Myocardial infarction	313 (20.5)	219 (19.6)
Cardiomyopathy	53 (3.5)	47 (4.2)
Any cardiac arrhythmia	636 (41.6)	490 (43.9)
Atrial fibrillation	523 (34.2)	399 (35.8)
Valvular heart disease	442 (28.9)	377 (33.8)
Cerebrovascular disease	71 (4.6)	72 (6.5)
Peripheral arterial disease	292 (19.1)	193 (17.3)
Hypertension	754 (49.3)	550 (49.3)
Dyslipidemia	476 (31.1)	315 (28.2)
Diabetes and associated complications	555 (36.3)	368 (33.0)
Major bleeding	183 (12.0)	118 (10.6)
Major intracranial hemorrhage	29 (1.9)	20 (1.8)
Major gastrointestinal bleeding	157 (10.3)	103 (9.2)
Chronic kidney disease	788 (51.5)	592 (53.1)
Chronic kidney disease (CrCl < 30 mL/min/m^2)	207 (13.5)	125 (11.2)
Acute kidney disease	287 (18.8)	248 (22.2)
Anemia	81 (5.3)	48 (4.3)
Chronic obstructive pulmonary disorder	549 (35.9)	394 (35.3)
Pulmonary edema	20 (1.3)	23 (2.1)
Pneumonia	92 (6.0)	80 (7.2)
Liver disease	23 (1.5)	24 (2.2)
Rheumatic disease	141 (9.2)	95 (8.5)
Gastroduodenal ulcers	27 (1.8)	31 (2.8)
Depression	32 (2.1)	40 (3.6)
Malign cancer	286 (18.7)	167 (15.0)

**Medical procedures at cohort entry**, n (%)		
Percutaneous coronary intervention – stent/CABG	144 (9.4)	62 (5.6)
Medical procedures for a defibrillator	73 (4.8)	52 (4.7)

Table S2. Demographics, clinical characteristics, and healthcare use of cases from nested case-control and excluded deaths.

SD, standard deviation; IQR, interquartile range; CABG, coronary artery bypass grafting; CrCl, creatinine clearance.

**Variables**	**All HF cohort (N = 12,242)**	**Left ventricular HF (n = 6,134)**	**Non-specific HF (n = 6,108)**
**Male**, n (%)	5,549 (45.3)	2,610 (42.6)	2,239 (48.1)
**Age** (in years)			
Mean ± SD	81.6 ± 6.9	81.5 ± 6.9	81.7 ± 6.9
Median (IQR)	81.5 (9.9)	81.4 (10.0)	81.6 (9.8)
**Group**, n (%)			
66–74	5,430 (44.4)	2,685 (43.8)	2,745 (44.9)
75–84	5,197 (42.4)	2,612 (42.6)	2,585 (42.3)
85 +	1,615 (13.2)	837 (13.6)	778 (12.7)
**Charlson score**, mean ± SD	5.2 ± 2.8	5.2 ± 2.8	5.2 ± 2.8
**Charlson score**, n (%)			
< 4	3,575 (29.2)	1,780 (29.0)	1,795 (29.4)
≥ 4	8,667 (70.8)	4,354 (71.0)	4,313 (70.6)
**Frailty score**, n (%)			
≤ -1	1 (0.0)	1 (0.0)	0 (0.0)
0–3	1,346 (11.0)	606 (9.9)	740 (12.0)
4–8	2,706 (22.1)	1,375 (22.4)	1,331 (21.8)
9–15	4,031 (32.9)	1,977 (32.2)	2,054 (33.7)
≥ 16	4,158 (34.0)	2,175 (35.5)	1,983 (32.5)
**Comorbidities at cohort entry**, n (%)			
Coronary artery disease (excluding myocardial infarction)	4,738 (38.7)	2,430 (39.6)	2,308 (37.8)
Myocardial infarction	2,258 (18.4)	1,296 (21.1)	962 (15.8)
Cardiomyopathy	618 (5.1)	272 (4.4)	346 (5.7)
Any cardiac arrhythmia	5,450 (44.5)	2,683 (43.7)	2,767 (45.3)
Atrial fibrillation	4,360 (35.6)	2,102 (34.3)	2,258 (37.0)
Valvular heart disease	3,239 (26.5)	1,657 (27.0)	1,582 (25.9)
Cerebrovascular disease	552 (4.5)	340 (5.5)	212 (3.5)
Peripheral artery disease	1,839 (15.0)	1,033 (16.8)	806 (13.2)
Hypertension	6,733 (55.0)	3,376 (55.0)	3,357 (55.0)
Dyslipidemia	4,455 (36.4)	2,232 (36.4)	2,223 (36.4)
Diabetes and associated complications	4,351 (35.5)	2,192 (35.7)	2,159 (35.4)
Major bleeding	1092 (8.9)	515 (8.4)	577 (9.5)
Major intracranial hemorrhage	162 (1.3)	76 (1.2)	86 (1.4)
Major gastrointestinal bleeding	952 (7.8)	449 (7.3)	503 (8.2)
Chronic kidney disease	5,099 (41.7)	2,570 (41.9)	2,529 (41.4)

Chronic kidney disease (CrCl < 30 mL/min/m^2)	1,172 (9.6)	532 (8.7)	640 (10.5)
Acute kidney disease	2,072 (16.9)	1,088 (17.7)	984 (16.1)
Anemia	507 (4.1)	251 (4.1)	256 (4.2)
Chronic obstructive pulmonary disorder	3,746 (30.6)	1,999 (32.6)	1,747 (28.6)
Pulmonary edema	145 (1.2)	40 (0.7)	105 (1.7)
Pneumonia	671 (5.5)	377 (6.2)	294 (4.8)
Liver disease	150 (1.2)	62 (1.0)	88 (1.4)
Rheumatic disease	958 (7.8)	443 (7.2)	515 (8.4)
Gastroduodenal ulcers	289 (2.4)	141 (2.3)	148 (2.4)
Depression	243 (2.0)	108 (1.8)	135 (2.2)
Malign cancer	1,990 (16.3)	946 (15.4)	1,044 (17.1)
**Medical procedures at cohort entry**, n (%)			
Percutaneous coronary intervention - stent	818 (6.7)	391 (6.4)	427 (7.0)
Coronary artery bypass grafting	392 (3.2)	175 (2.9)	217 (3.6)
Medical procedures for a defibrillator	553 (4.5)	241 (3.9)	312 (5.1)
**CVD drug use (three-month prior the case or control selection)**, n (%)			
Diuretics	7,885 (64.4)	3,754 (61.2)	4,131 (67.6)
Loop diuretics	6,850 (56.0)	3,186 (51.9)	3,664 (60.0)
Metolazone	75 (0.6)	20 (0.3)	55 (0.9)
ACEIs/ARBs	6,947 (56.8)	3,456 (56.3)	3,491 (57.2)
ACEIs	4,666 (38.1)	2,370 (38.6)	2,296 (37.6)
ARBs	2,540 (20.8)	1,220 (19.9)	1,320 (21.6)
Beta-blockers	6,770 (55.3)	3,328 (54.3)	3,442 (56.4)
Metoprolol	3,080 (25.2)	1,529 (24.9)	1,551 (25.4)
Carvedilol	318 (2.6)	117 (1.9)	201 (3.3)
Bisoprolol	2,452 (20.0)	1,198 (19.5)	1,254 (20.5)
Other beta-blockers	1134 (9.3)	585 (9.5)	549 (9.0)
Spironolactone or eplerenone	753 (6.2)	290 (4.7)	463 (7.6)
Digoxin	1,874 (15.3)	809 (13.2)	1,065 (17.4)
Hydralazine	211 (1.7)	110 (1.8)	101 (1.7)
Nitrates	3,889 (31.8)	2,069 (33.7)	1,820 (29.8)
Statin	6,637 (54.2)	3,301 (53.8)	3,336 (54.6)
Antiarrhythmic (amiodarone or propafenone)	966 (7.9)	494 (8.1)	472 (7.7)
Warfarin	4,322 (35.3)	1,957 (31.9)	2,365 (38.7)
DOAC	497 (4.1)	222 (3.6)	275 (4.5)
Antiplatelets agents (without low-dose ASA)	1,674 (13.7)	890 (14.5)	784 (12.8)
Low-dose ASA	6,357 (51.9)	3,341 (54.5)	3,016 (49.4)

**Discharge drug (in one-month following discharge)**, n (%)			
ACEIs/ARBs	7,628 (62.3)	3,856 (62.9)	3,772 (61.8)
Beta-blockers	7,698 (62.9)	3,850 (62.8)	3,848 (63.0)
Spironolactone or eplerenone	1,898 (15.5)	724 (11.8)	1,174 (19.2)
**Antidiabetic agents (three-month prior the case or control selection)**, n (%)	3,986 (32.6)	1,996 (32.5)	1,990 (32.6)
Metformin	2,411 (19.7)	1,197 (19.5)	1,214 (19.9)
Sulfonylurea	1,837 (15.0)	931 (15.2)	906 (14.8)
Thiazolidinediones	280 (2.3)	150 (2.5)	130 (2.1)
DPP-4 inhibitors	203 (1.7)	94 (1.5)	109 (1.8)
SGLT2 inhibitors	0	0	0
GLP-1 agonists	0	0	0
Insulins	1,135 (9.3)	556 (9.1)	579 (9.5)
**Potentially inappropriate drug for HF exacerbation (three-month prior the case or control selection)**, n (%)			
Metformin	2,411 (19.7)	1,197 (19.5)	1,214 (19.9)
Sulfonylurea	1,837 (15.0)	931 (15.2)	906 (14.8)
Thiazolidinediones	280 (2.3)	150 (2.5)	130 (2.1)
DPP-4 inhibitors (saxagliptin/sitagliptin)	188 (1.5)	94 (1.5)	109 (1.8)
Calcium channel blockers	5,751 (47.0)	3,026 (49.3)	2,725 (44.6)
Diltiazem/verapamil	1,697 (13.9)	895 (14.6)	802 (13.1)
Nifedipine	829 (6.8)	467 (7.6)	362 (5.9)
Others	3,498 (28.6)	1,819 (29.7)	1,679 (27.5)
Carbamazepine	73 (0.6)	37 (0.6)	36 (0.6)
Citalopram or escitalopram	750 (6.1)	388 (6.3)	362 (5.9)
Clozapine	0	0	0
NSAIDs	811 (6.6)	467 (7.6)	344 (5.6)
Salbutamol	2,275 (18.6)	1,161 (18.9)	1,114 (18.2)
Hydroxychloroquine	118 (1.0)	60 (1.0)	58 (1.0)
**Other drug use (three-month prior the case or control selection)**, n (%)			
Proton pump inhibitors	5,272 (43.1)	2,624 (42.8)	2,648 (43.4)
Antidepressants agents	2,304 (18.8)	1,173 (19.1)	1,131 (18.5)
Anticholinergics agents	239 (2.0)	112 (1.8)	127 (2.1)
Benzodiazepine	4,612 (37.7)	2,421 (39.5)	2,191 (35.9)
**Health service**, n (%)			
All-cause hospital admission one-year prior cohort entry	6,316 (51.6)	3,133 (51.1)	3,183 (52.1)

Table S3. Demographic, clinical characteristics, and healthcare use of patients selected in the cohort with a diagnosis of all, non-specific HF and left ventricular HF.

HF, heart failure; SD, standard deviation; IQR, interquartile range; CVD, cardiovascular disease; CrCl, creatinine clearance; ACEIs, angiotensin-converting enzyme inhibitors; ARBs, angiotensin II receptor blockers; ASA, acetylsalicylic acid; DOAC, direct oral anticoagulant; DPP-4, dipeptidyl peptidase 4; GLP-1, glucagon-like peptide-1; NSAIDs, non-steroidal anti-inflammatory drugs; SGLT2, sodium glucose transport 2; Antiplatelets: clopidogrel, ticlopidine, prasugrel, ticagrelor.

	Crude RR (95% CI)	Adjusted RR (95% CI)
**Cardiovascular polypharmacy**		
< 5	reference	reference
≥ 5	0.79 (0.70–0.88)	0.97 (0.82–1.15)
**Non-cardiovascular polypharmacy**		
< 5	reference	reference
≥ 5	1.31 (1.16–1.47)	0.93 (0.77–1.12)
**Hyperpolypharmacy**		
< 10	reference	reference
≥ 10	1.27 (1.13–1.43)	1.31 (1.07–1.61)
**Type of HF**		
Ventricular HF	reference	reference
Non-specific HF	1.14 (1.03–1.27)	1.15 (1.03–1.28)
**Charlson score**		
< 4	reference	reference
≥ 4	1.67 (1.47–1.90)	1.20 (1.02–1.41)
**Frailty score**		
< 4	reference	reference
≥ 4–8	1.24 (0.99–1.55)	1.13 (0.89–1.44)
≥ 9–15	1.45 (1.17–1.79)	1.18 (0.93–1.50)
≥ 16	1.97 (1.60–2.43)	1.32 (1.03–1.69)
**Comorbidites at cohort entry (yes vs no)**		
Coronary artery disease (excluding myocardial infarction)	1.08 (0.97–1.21)	1.08 (0.95–1.24)
Myocardial infarction	1.12 (0.99–1.28)	1.19 (1.01–1.40)
Cardiomyopathy	0.70 (0.53–0.93)	0.71 (0.53–0.95)
Any cardiac arrhythmia	0.87 (0.78–0.96)	0.78 (0.63–0.96)
Atrial fibrillation	0.93 (0.83–1.04)	1.20 (0.96–1.50)
Valvular heart disease	1.16 (1.03–1.30)	1.15 (1.02–1.30)
Cerebrovascular disease	1.10 (0.86–1.42)	0.98 (0.76–1.27)
Peripheral artery disease	1.41 (1.23–1.61)	1.30 (1.13–1.51)
Hypertension	0.74 (0.67–0.83)	0.86 (0.77–0.96)
Dyslipidemia	0.77 (0.69–0.86)	0.85 (0.74–0.97)
Diabetes and associated complications	1.09 (0.97–1.21)	1.20 (1.01–1.44)
Major bleeding	1.48 (1.25–1.74)	1.23 (1.04–1.47)
Chronic kidney disease	1.54 (1.39–1.71)	1.30 (1.13–1.49)
Chronic kidney disease (CrCl < 30 mL/min/m^2)	1.59 (1.36–1.86)	1.38 (1.16–1.64)
Acute kidney disease	1.19 (1.04–1.36)	0.96 (0.82–1.12)
Anemia	1.29 (1.02–1.64)	1.00 (0.78–1.29)
Chronic obstructive pulmonary disorder	1.36 (1.22–1.52)	1.06 (0.93–1.21)
Pneumonia	1.18 (0.95–1.48)	1.09 (0.87–1.38)
Liver disease	1.76 (1.13–2.76)	1.52 (0.96–2.42)
Depression	1.17 (0.81–1.70)	0.96 (0.65–1.41)
Malign cancer	1.13 (0.99–1.30)	1.05 (0.91–1.21)
**Medical procedures at cohort entry**		
Percutaneous coronary intervention – stent/CABG	1.04 (0.87–1.25)	1.00 (0.83–1.22)
Medical procedures for a defibrillator	1.01 (0.79–1.29)	0.94 (0.73–1.21)
**CVD drug use (three-month prior the case or control selection)**		
Diuretics	0.90 (0.75–1.08)	0.61 (0.32–1.16)
Loop diuretics	0.98 (0.83–1.17)	1.51 (0.81–2.80)
ACEIs/ARBs	0.64 (0.55–0.75)	0.76 (0.65–0.89)

Beta-blockers	0.74 (0.66–0.82)	0.78 (0.69–0.89)
Spironolactone or eplerenone	1.25 (1.09–1.42)	1.21 (1.05–1.40)
Digoxin	1.02 (0.90–1.16)	0.99 (0.86–1.14)
Hydralazine	2.08 (1.68–2.58)	1.63 (1.30–2.05)
Nitrates	1.27 (1.14–1.41)	1.14 (1.00–1.29)
Statins	0.68 (0.62–0.76)	0.68 (0.60–0.78)
Antiarrhythmic (amiodarone/propafenone)	1.18 (1.00–1.40)	1.16 (0.97–1.39)
Warfarin or DOAC	0.79 (0.71–0.88)	0.76 (0.66–0.88)
Antiplatelets (including ASA)	0.95 (0.86–1.06)	0.86 (0.74–0.98)
Antidiabetics	0.95 (0.84–1.06)	0.85 (0.68–1.06)
**Potentially inappropriate drug for HF exacerbation (three-month prior the case or control selection)**		
Metformin	0.86 (0.74–1.01)	1.10 (0.90–1.35)
Sulfonylurea	0.85 (0.72–0.99)	0.88 (0.72–1.08)
Thiazolidinediones	1.14 (0.55–2.37)	1.38 (0.64–2.94)
DPP-4 inhibitors (saxagliptin/sitagliptin)	0.70 (0.45–1.08)	0.73 (0.47–1.15)
Calcium channel blockers	0.77 (0.69–0.86)	0.76 (0.43–1.36)
Diltiazem/verapamil	0.85 (0.79–1.13)	0.99 (0.57–1.73)
Nifedipine	0.61 (0.43–0.86)	0.74 (0.39–1.39)
Others	0.79 (0.70–0.90)	0.97 (0.55–1.71)
Carbamazepine	1.44 (0.68–3.03)	1.47 (0.69–3.12)
Citalopram or escitalopram	1.28 (1.06–1.54)	0.97 (0.67–1.22)
NSAIDs	0.54 (0.36–0.80)	0.51 (0.34–0.76)
Salbutamol	1.51 (1.33–1.91)	1.25 (1.09–1.44)
Hydroxychlroroquine	1.46 (0.92–2.32)	1.55 (0.96–2.50)
**Other drug use (three-month prior the case or control selection)**		
Proton pump inhibitors	1.11 (0.99–1.23)	0.99 (0.88–1.12)
Antidepressants agents	1.37 (1.21–1.55)	1.25 (1.07–1.47)
Anticholinergics agents	1.54 (1.11–2.14)	1.32 (0.94–1.86)
Benzodiazepine	1.23 (1.11–1.37)	1.11 (0.99–1.24)

Table S4. Rate ratios (RR; 95% confidence interval (CI)) for the associations between polypharmacy in the last three months and all-cause mortality.

RR, rate ratio; CI, confidence interval; CVD, cardiovascular disease; CABG, coronary artery bypass grafting; CrCl, creatinine clearance; ACEIs, angiotensin-converting enzyme inhibitors; ARBs, angiotensin II receptor blockers; HF, heart failure; DOAC, direct oral anticoagulant; DPP-4, dipeptidyl peptidase 4; NSAIDs, non-steroidal anti-inflammatory drugs; ASA, acetylsalicylic acid; Antiplatelets: clopidogrel, ticlopidine, prasugrel, ticagrelor.

Variables	All HF cohort (N = 12,242)	Left ventricular HF (n = 6,134)	Non-specific HF (n = 6,108)
**Male**, n (%)	5,549 (45.3)	2,610 (42.6)	2,239 (48.1)
**Age (in years)**			
Mean ± SD	81.6 ± 6.9	81.5 ± 6.9	81.7 ± 6.9
Median (IQR)	81.5 (9.9)	81.4 (10.0)	81.6 (9.8)
**Group**, n (%)			
66–74	5,430 (44.4)	2,685 (43.8)	2,745 (44.9)
75–84	5,197 (42.4)	2,612 (42.6)	2,585 (42.3)
85 +	1,615 (13.2)	837 (13.6)	778 (12.7)
**Charlson-Deyo score**, mean ± SD	5.2 ± 2.8	5.2 ± 2.8	5.2 ± 2.8
**Charlson-Deyo score**, n (%)			
< 4	3,575 (29.2)	1,780 (29.0)	1,795 (29.4)
≥ 4	8,667 (70.8)	4,354 (71.0)	4,313 (70.6)
**Frailty score**, n (%)			
Robust (Frailty score ≤ -1)	1 (0.0)	1 (0.0)	0 (0.0)
Well (Frailty score: 0–3)	1,346 (11.0)	606 (9.9)	740 (12.0)
Well/comorbidities (Frailty score: 4–8)	2,706 (22.1)	1,375 (22.4)	1,331 (21.8)
Pre-frail (Frailty score: 9–15)	4,031 (32.9)	1,977 (32.2)	2,054 (33.7)
Frail (Frailty score: ≥ 16)	4,158 (34.0)	2,175 (35.5)	1,983 (32.5)
**Comorbidities (one-year prior cohort entry)**, n (%)			
Coronary artery disease (excluding myocardial infarction)	5,392 (44.1)	2,597 (42.3)	2,795 (45.8)
Myocardial infarction	4,283 (35.0)	2,314 (37.7)	1,969 (32.2)
Cardiomyopathy	1,279 (10.5)	564 (9.2)	715 (11.7)
Any cardiac arrhythmia	8,405 (68.7)	4,078 (66.5)	4,327 (70.8)
Atrial fibrillation	6,287 (51.4)	2,965 (48.3)	3,322 (54.4)
Valvular heart disease	4,649 (38.0)	2,309 (37.6)	2,340 (38.3)
Cerebrovascular disease	2,513 (20.5)	1,305 (21.3)	1,208 (19.8)
Peripheral artery disease	3,651 (29.8)	1,868 (30.5)	1,783 (29.2)
Hypertension	10,020 (81.9)	5,090 (83.0)	4,930 (80.7)
Dyslipidemia	5,986 (48.9)	3,000 (48.9)	2,986 (48.9)
Diabetes and associated complications	5,324 (43.5)	2,652 (43.2)	2,672 (43.8)
Major bleeding	1,092 (8.9)	515 (8.4)	577 (9.5)
Major intracranial hemorrhage	162 (1.3)	76 (1.2)	86 (1.4)
Major gastrointestinal bleeding	952 (7.8)	449 (7.3)	503 (8.2)
Chronic kidney disease	5,099 (41.7)	2,570 (41.9)	2,529 (41.4)

Chronic kidney disease (CrCl < 30 mL/min/m^2)	1,172 (9.6)	532 (8.7)	640 (10.5)
Acute kidney disease	3,539 (28.9)	1,813 (29.6)	1,726 (28.3)
Anemia	507 (4.1)	251 (4.1)	256 (4.2)
Chronic obstructive pulmonary disorder	5,935 (48.5)	3,078 (50.2)	2,857 (46.8)
Pneumonia	3,827 (31.3)	1,973 (32.2)	1,854 (30.4)
Liver disease	350 (2.9)	140 (2.3)	210 (3.4)
Rheumatic disease	1,786 (14.6)	802 (13.1)	984 (16.1)
Gastroduodenal ulcers	1,684 (13.8)	865 (14.1)	819 (13.4)
Depression	1,455 (11.9)	699 (11.4)	756 (12.4)
Malign Cancer	1,536 (12.6)	716 (11.7)	820 (13.4)
**Medical procedures (one-year prior cohort entry)**, n (%)			
Percutaneous coronary intervention – stent	818 (6.7)	391 (6.4)	427 (7.0)
Coronary artery bypass grafting	392 (3.2)	175 (2.9)	217 (3.6)
Medical procedures for a defibrillator	553 (4.5)	241 (3.9)	312 (5.1)
**CVD drug use (three-month prior the case or control selection)**, n (%)			
Diuretics	7,885 (64.4)	3,754 (61.2)	4,131 (67.6)
Loop diuretics	6,850 (56.0)	3,186 (51.9)	3,664 (60.0)
Metolazone	75 (0.6)	20 (0.3)	55 (0.9)
Angiotensin II inhibitors	6,947 (56.8)	3,456 (56.3)	3,491 (57.2)
ACE inhibitors	4,666 (38.1)	2,370 (38.6)	2,296 (37.6)
Angiotensin II receptor blockers	2,540 (20.8)	1,220 (19.9)	1,320 (21.6)
Beta-blockers	6,770 (55.3)	3,328 (54.3)	3,442 (56.4)
Metoprolol	3,080 (25.2)	1,529 (24.9)	1,551 (25.4)
Carvedilol	318 (2.6)	117 (1.9)	201 (3.3)
Bisoprolol	2,452 (20.0)	1,198 (19.5)	1,254 (20.5)
Other Beta-blockers	1134 (9.3)	585 (9.5)	549 (9.0)
Spironolactone or eplerenone	753 (6.2)	290 (4.7)	463 (7.6)
Digoxine	1,874 (15.3)	809 (13.2)	1,065 (17.4)
Hydralazine	211 (1.7)	110 (1.8)	101 (1.7)
Nitrates	3,889 (31.8)	2,069 (33.7)	1,820 (29.8)
Statin	6,637 (54.2)	3,301 (53.8)	3,336 (54.6)
Antiarrhythmic (amiodarone or propafenone)	966 (7.9)	494 (8.1)	472 (7.7)
Warfarin	4,322 (35.3)	1,957 (31.9)	2,365 (38.7)
DOAC	497 (4.1)	222 (3.6)	275 (4.5)
Antiplatelets agents (without low-dose ASA)	1,674 (13.7)	890 (14.5)	784 (12.8)
Low-dose ASA	6,357 (51.9)	3,341 (54.5)	3,016 (49.4)
**Antidiabetic agents (three-month prior the case or control selection)**, n (%)			

Metformin	2,411 (19.7)	1,197 (19.5)	1,214 (19.9)
Sulfonylurea	1,837 (15.0)	931 (15.2)	906 (14.8)
Thiazolidinediones	280 (2.3)	150 (2.5)	130 (2.1)
DPP-4 inhibitors	203 (1.7)	94 (1.5)	109 (1.8)
SGLT-2 inhibitors	0	0	0
GLP-1 agonists	0	0	0
Insulins	1,135 (9.3)	556 (9.1)	579 (9.5)
**Potentially inappropriate drug for HF exacerbation (three-month prior the case or control selection**, n (%)			
Antidiabetic agents			
Metformin	2,411 (19.7)	1,197 (19.5)	1,214 (19.9)
Sulfonylurea	1,837 (15.0)	931 (15.2)	906 (14.8)
Thiazolidinediones	280 (2.3)	150 (2.5)	130 (2.1)
DPP-4 inhibitors (saxagliptin/sitagliptin)	188 (1.5)	94 (1.5)	109 (1.8)
Calcium channel blockers	5,751 (47.0)	3,026 (49.3)	2,725 (44.6)
Diltiazem/verapamil	1,697 (13.9)	895 (14.6)	802 (13.1)
Nifedipine	829 (6.8)	467 (7.6)	362 (5.9)
Others	3,498 (28.6)	1,819 (29.7)	1,679 (27.5)
Carbamazepine	73 (0.6)	37 (0.6)	36 (0.6)
Citalopram or escitalopram	750 (6.1)	388 (6.3)	362 (5.9)
Clozapine	0	0	0
NSAIDs	811 (6.6)	467 (7.6)	344 (5.6)
Salbutamol	2,275 (18.6)	1,161 (18.9)	1,114 (18.2)
Hydroxychloroquine	118 (1.0)	60 (1.0)	58 (1.0)
**Other drug use (three-month prior the case or control selection)**, n (%)			
Proton pump inhibitors	5,272 (43.1)	2,624 (42.8)	2,648 (43.4)
Antidepressants agents	2,304 (18.8)	1,173 (19.1)	1,131 (18.5)
Anticholinergics agents	239 (2.0)	112 (1.8)	127 (2.1)
Benzodiazepine	4,612 (37.7)	2,421 (39.5)	2,191 (35.9)
**Health service**, n (%)			
All-cause hospital admission one-year prior cohort entry	6,316 (51.6)	3,133 (51.1)	3,183 (52.1)

Table S5. Demographic, clinical characteristics, and healthcare use of patients with a diagnosis of all, non-specific HF and left ventricular HF.

HF, heart failure; SD, standard deviation; IQR, interquartile range; CVD, cardiovascular disease; ACE, angiotensin-converting enzyme; ASA, acetylsalicylic acid; DOAC, direct oral anticoagulant; NSAIDs, non-steroidal anti-inflammatory drugs; Antiplatelets: clopidogrel, ticlopidine, prasugrel, ticagrelor.

	Crude RR (95% CI)		Adjusted RR (95% CI)	
	Secondary diagnosis during HF hospitalization	Diagnosis in one-year prior and during HF hospitalization	Secondary diagnosis during HF hospitalization	Diagnosis in one-year prior and during HF hospitalization
**Cardiovascular polypharmacy**				
< 5	reference	reference	reference	reference
≥ 5	0.79 (0.70–0.88)	0.79 (0.70–0.88)	0.97 (0.82–1.15)	0.95 (0.80–1.13)
**Non-cardiovascular polypharmacy**				
< 5	reference	reference	reference	reference
≥ 5	1.31 (1.16–1.47)	1.31 (1.16–1.47)	0.93 (0.77–1.12)	0.93 (0.77–1.12)
**Hyperpolypharmacy**				
< 10	reference	reference	reference	reference
≥ 10	1.27 (1.13–1.43)	1.27 (1.13–1.43)	1.31 (1.07–1.61)	1.33 (1.08–1.63)
**Type of HF**				
Ventricular HF	reference	reference	reference	reference
Non-specific HF	1.14 (1.03–1.27)	1.14 (1.03–1.27)	1.15 (1.03–1.28)	1.14 (1.02–1.27)
**Charlson score**				
< 4	reference	reference	reference	reference
≥ 4	1.67 (1.47–1.90)	1.67 (1.47–1.90)	1.20 (1.02–1.41)	1.09 (0.92–1.29)
**Frailty score**				
< 4	reference	reference	reference	reference
≥ 4–8	1.24 (0.99–1.55)	1.24 (0.99–1.55)	1.13 (0.89–1.44)	1.14 (0.90–1.45)
≥ 9–15	1.45 (1.17–1.79)	1.45 (1.17–1.79)	1.18 (0.93–1.50)	1.17 (0.92–1.48)
≥ 16	1.97 (1.60–2.43)	1.97 (1.60–2.43)	1.32 (1.03–1.69)	1.30 (1.01–1.67)
**Comorbidites (yes vs no)**				
Coronary artery disease (excluding myocardial infarction)	1.08 (0.97–1.21)	0.98 (0.88–1.09)	1.08 (0.95–1.24)	1.09 (0.94–1.27)
Myocardial infarction	1.12 (0.99–1.28)	1.23 (1.10–1.38)	1.19 (1.01–1.40)	1.23 (1.03–1.46)
Cardiomyopathy	0.70 (0.53–0.93)	1.07 (0.89–1.29)	0.71 (0.53–0.95)	1.01 (0.83–1.23)
Any cardiac arrhythmia	0.87 (0.78–0.96)	0.89 (0.80–0.99)	0.78 (0.63–0.96)	0.85 (0.72–1.01)
Atrial fibrillation	0.93 (0.83–1.04)	0.93 (0.84–1.04)	1.20 (0.96–1.50)	1.09 (0.92–1.30)
Valvular heart disease	1.16 (1.03–1.30)	1.23 (1.10–1.37)	1.15 (1.02–1.30)	1.18 (1.06–1.33)
Cerebrovascular disease	1.10 (0.86–1.42)	1.24 (1.07–1.44)	0.98 (0.76–1.27)	1.10 (0.94–1.29)
Peripheral artery disease	1.41 (1.23–1.61)	1.37 (1.21–1.54)	1.30 (1.13–1.51)	1.23 (1.08–1.39)
Hypertension	0.74 (0.67–0.83)	0.85 (0.75–0.95)	0.86 (0.77–0.96)	0.91 (0.80–1.03)
Dyslipidemia	0.77 (0.69–0.86)	0.86 (0.77–0.96)	0.85 (0.74–0.97)	0.89 (0.79–1.02)
Diabetes and associated complications	1.09 (0.97–1.21)	1.11 (0.99–1.23)	1.20 (1.01–1.44)	1.17 (0.99–1.39)

Major bleeding (three years prior)	1.48 (1.25–1.74)	1.48 (1.25–1.74)	1.23 (1.04–1.47)	1.23 (1.03–1.46)
Chronic kidney disease	1.54 (1.39–1.71)	1.66 (1.49–1.85)	1.30 (1.13–1.49)	1.41 (1.23–1.63)
Chronic kidney disease (CrCl < 30 mL/min/m^2)	1.59 (1.36–1.86)	1.59 (1.36–1.86)	1.38 (1.16–1.64)	1.32 (1.11–1.57)
Acute kidney disease	1.19 (1.04–1.36)	1.39 (1.24–1.56)	0.96 (0.82–1.12)	1.01 (0.88–1.16)
Anemia	1.29 (1.02–1.64)	1.29 (1.02–1.64)	1.00 (0.78–1.29)	1.00 (0.78–1.28)
Chronic obstructive pulmonary disorder	1.36 (1.22–1.52)	1.34 (1.21–1.49)	1.06 (0.93–1.21)	1.06 (0.93–1.20)
Pneumonia	1.18 (0.95–1.48)	1.21 (1.07–1.37)	1.09 (0.87–1.38)	1.01 (0.88–1.15)
Liver disease	1.76 (1.13–2.76)	1.56 (1.11–2.18)	1.52 (0.96–2.42)	1.23 (0.87–1.75)
Depression	1.17 (0.81–1.70)	1.08 (0.88–1.32)	0.96 (0.65–1.41)	0.84 (0.68–1.05)
Malign cancer (three years prior)	1.16 (0.99–1.34)	1.16 (0.99–1.34)	1.05 (0.91–1.21)	1.09 (0.93–1.28)
**Medical procedures**, n (%)				
Percutaneous coronary intervention – stent/CABG	1.04 (0.87–1.25)	1.04 (0.87–1.25)	1.00 (0.83–1.22)	0.97 (0.79–1.18)
Medical procedures for a defibrillator	1.01 (0.79–1.29)	1.01 (0.79–1.29)	0.94 (0.73–1.21)	0.94 (0.73–1.22)
**CVD drug use (three-month prior the case or control selection)**				
Diuretics	0.90 (0.75–1.08)	0.90 (0.75–1.08)	0.61 (0.32–1.16)	0.61 (0.32–1.15)
Loop diuretics	0.98 (0.83–1.17)	0.98 (0.83–1.17)	1.51 (0.81–2.80)	1.54 (0.83–2.85)
ACEIs/ARBs	0.64 (0.55–0.75)	0.64 (0.55–0.75)	0.76 (0.65–0.89)	0.75 (0.65–0.88)
Beta-blockers	0.74 (0.66–0.82)	0.74 (0.66–0.82)	0.78 (0.69–0.89)	0.77 (0.68–0.87)
Spironolactone or eplerenone	1.25 (1.09–1.42)	1.25 (1.09–1.42)	1.21 (1.05–1.40)	1.20 (1.04–1.38)
Digoxine	1.02 (0.90–1.16)	1.02 (0.90–1.16)	0.99 (0.86–1.14)	0.99 (0.85–1.14)
Hydralazine	2.08 (1.68–2.58)	2.08 (1.68–2.58)	1.63 (1.30–2.05)	1.61 (1.28–2.03)
Nitrates	1.27 (1.14–1.41)	1.27 (1.14–1.41)	1.14 (1.00–1.29)	1.14 (1.00–1.29)
Statins	0.68 (0.62–0.76)	0.68 (0.62–0.76)	0.68 (0.60–0.78)	0.68 (0.59–0.78)
Antiarrhythmic (amiodarone or propafenone)	1.18 (1.00–1.40)	1.18 (1.00–1.40)	1.16 (0.99–1.39)	1.13 (0.94–1.36)
Warfarin or DOAC	0.79 (0.71–0.88)	0.79 (0.71–0.88)	0.76 (0.66–0.88)	0.76 (0.65–0.88)
Antiplatelets (including ASA)	0.95 (0.86–1.06)	0.95 (0.86–1.06)	0.86 (0.74–0.98)	0.86 (0.74–0.98)
**Potentially inappropriate drug for HF exacerbation (three-month prior the case or control selection)**, n (%)				
Antidiabetic agents	0.95 (0.84–1.06)	0.95 (0.84–1.06)	0.85 (0.68–1.06)	0.86 (0.69–1.07)
Metformin	0.86 (0.74–1.00)	0.86 (0.74–1.00)	1.10 (0.90–1.35)	1.12 (0.92–1.37)
Sulfonylurea	0.85 (0.72–0.99)	0.85 (0.72–0.99)	0.88 (0.72–1.08)	0.89 (0.73–1.08)
Thiazolidinediones	1.14 (0.55–2.37)	1.14 (0.55–2.37)	1.38 (0.64–2.94)	1.41 (0.66–3.02)
DPP-4 inhibitors	0.70 (0.45–1.08)	0.70 (0.45–1.08)	0.73 (0.47–1.15)	0.73 (0.46–1.14)
Calcium channel blockers	0.77 (0.69–0.86)	0.77 (0.69–0.86)	0.76 (0.43–1.36)	0.76 (0.42–1.35)
Diltiazem/verapamil	0.95 (0.79–1.13)	0.95 (0.79–1.13)	0.99 (0.57–1.73)	1.00 (0.57–1.75)
Nifedipine	0.61 (0.43–0.86)	0.61 (0.43–0.86)	0.74 (0.39–1.39)	0.74 (0.40–1.40)
Other CCBs	0.79 (0.70–0.90)	0.79 (0.70–0.90)	0.97 (0.55–1.71)	0.98 (0.56–1.73)
Carbamazepine	1.44 (0.68–3.03)	1.44 (0.68–3.03)	1.47 (0.69–3.12)	1.46 (0.68–3.11)

Citalopram or escitalopram	1.28 (1.06–1.54)	1.28 (1.06–1.54)	0.97 (0.77–1.22)	0.96 (0.77–1.21)
NSAIDs	0.54 (0.36–0.80)	0.54 (0.36–0.80)	0.51 (0.34–0.76)	0.53 (0.35–0.78)
Salbutamol	1.51 (1.33–1.71)	1.51 (1.33–1.71)	1.25 (1.09–1.44)	1.27 (1.10–1.47)
Hydroxychloroquine	1.46 (0.92–2.32)	1.46 (0.92–2.32)	1.55 (0.96–2.50)	1.55 (0.96–2.49)
**Other drug use (three-month prior the case or control selection)**, n (%)				
Proton pump inhibitors	1.11 (0.99–1.23)	1.11 (0.99–1.23)	0.99 (0.88–1.12)	0.99 (0.87–1.11)
Antidepressants agents	1.37 (1.21–1.55)	1.37 (1.21–1.55)	1.25 (1.07–1.47)	1.28 (1.09–1.51)
Anticholinergics agents	1.54 (1.11–2.14)	1.54 (1.11–2.14)	1.32 (0.94–1.86)	1.30 (0.92–1.82)
Benzodiazepine	1.23 (1.11–1.37)	1.23 (1.11–1.37)	1.11 (0.99–1.24)	1.11 (0.99–1.24)

Table S6. Rate ratios (RR; 95% confidence interval (CI)) for the associations between polypharmacy in the last three months and all-cause mortality.

RR, rate ratio; CI, confidence interval; CVD, cardiovascular disease; CrCl, creatinine clearance; CABG, coronary artery bypass grafting; ACEIs, angiotensin-converting enzyme inhibitors; ARBs, angiotensin II receptor blockers; HF, heart failure; DOAC, direct oral anticoagulant; ASA, acetylsalicylic acid; Antiplatelets: clopidogrel, ticlopidine, prasugrel, ticagrelor.
